# Treatment outcomes and survival analysis of pediatric mature B-Cell non-Hodgkin lymphoma: A retrospective study comparing LMB96 and R-CHOP regimens

**DOI:** 10.1016/j.lrr.2025.100531

**Published:** 2025-07-23

**Authors:** Hamid D. Habeeb Rjeib, Usama Al-Jumaily, Inas Muayad Mohammed Ali, Safa Faraj, Mohammed Fawzi Al-Qanbar, Dheyaa Aldeen Al-Khateeb, Shaima Jabbar

**Affiliations:** a Department.of Pathology, College of Medicine, University of Al-Qadisiyah, Al-Qadisiyah, Iraq; b Al-Subtain University of Medical Sciences/TUMS, Karbala, Iraq; c Department.of Pediatrics, College of Medicine, University of Kerbala, Karbala, Iraq; d Department of Pediatrics, College of Medicine, Wasit University, Wasit, Iraq; e Department of Pathology, College of Medicine, University of Kerbala, Karbala, Iraq; f Al-Subtain University of Medical Sciences/ College of Medicine/TUMS, Karbala, Iraq; g Public health Department, Alhassan Al-Mojtaba hospital, Kerbala health director, Karbala, Iraq; h Departmeent of Pharmacology and Toxicology, College of Pharmacy, University of Kerbala, Karbala, Iraq

**Keywords:** B–cell non–Hodgkin lymphoma (B–NHL), Pediatric lymphoma, Burkitt's lymphoma, LMB96 protocol, R–CHOP regimen, Event–free survival (EFS)

## Abstract

**Background:**

Mature B-cell non-Hodgkin lymphoma (B-NHL) is a prevalent pediatric malignancy with significant treatment advancements. This study retrospectively analyzes clinical characteristics, treatment outcomes, and survival rates of children and adolescents diagnosed with B-NHL at Al-Hasan Al-Mojtaba Hospital between January 2014 and December 2024. A comparative analysis was performed between the LMB96 and R-CHOP regimens.

**Methods:**

Patients with confirmed diagnoses of Large B-cell lymphoma or Burkitt’s lymphoma, based on WHO classification criteria, were included. Staging was conducted using the St. Jude system, and risk classification followed the FAB/LMB criteria. Treatment involved a modified LMB96 regimen, later replaced by R-CHOP in the last two years of the study. Event-free survival (EFS) was analyzed using Kaplan-Meier survival curves, with stratifications by staging, risk group, and gender.

**Results:**

A total of 66 patients were included (median age: 5.8 years; 69.7 % male). Burkitt’s lymphoma was the predominant histology (86.3 %). The abdomen was the most common primary site (84.8 %). The majority of patients (72.7 %) presented with advanced-stage disease (Stages III and IV). Risk group classification identified 62.1 % of patients in Group B and 28.8 % in Group C. Kaplan-Meier survival analysis revealed Group A had the most favorable prognosis (EFS ∼100 %), followed by Group B (∼75 %), and Group C (∼50 %). Disease stage significantly influenced survival (*p* = 0.021), with Stage IV patients demonstrating the poorest outcomes. While female patients exhibited higher EFS than males, the difference was not statistically significant (*p* = 0.27). By the end of follow-up, 28.8 % of patients had experienced a fatal outcome.

**Conclusion:**

Advanced-stage B-NHL remains prevalent, with significant survival differences based on staging and risk classification. The transition from LMB96 to R-CHOP warrants further evaluation to optimize pediatric treatment strategies. Larger studies are needed to validate observed gender-based survival trends.

## 1 Introduction

Mature B-cell non-Hodgkin lymphoma (B-NHL) is an aggressive subtype of pediatric lymphoma, accounting for a significant proportion of childhood cancers. Despite advancements in chemotherapy regimens, survival outcomes in children remain inferior compared to adult patients. The 5-year overall survival (OS) rate for pediatric B-NHL is approximately 60 %, whereas adult patients achieve over 90 % survival at the same timepoint. This disparity underscores the need for improved treatment strategies and supportive care measures, particularly in resource-limited settings [1,2].

Pediatric B-NHL encompasses a heterogeneous group of lymphoid malignancies, with Burkitt lymphoma (BL) and diffuse large B-cell lymphoma (DLBCL) being the most prevalent subtypes. BL is further classified into sporadic, endemic, and immunodeficiency-associated forms, all of which share characteristic genetic alterations, including the t(8;14) translocation involving the MYC oncogene. The aggressive nature of B-NHL necessitates intensive, short-duration chemotherapy protocols, such as the LMB96 and BFM regimens, which have significantly improved survival in high-income countries. However, outcomes in low- and middle-income countries (LMICs) remain suboptimal due to late-stage diagnoses, treatment-related toxicities, and limited access to supportive care [[3], [4], [5]].

Over the past few decades, several clinical trials have refined treatment strategies for pediatric B-NHL. Studies have shown that dose-intensive chemotherapy regimens, including high-dose methotrexate (Methotrexate) for central nervous system (CNS) prophylaxis, contribute to improved progression-free survival (PFS) rates of 70 %–90 %. The incorporation of rituximab, an anti-CD20 monoclonal antibody, has further enhanced outcomes, particularly in patients with primary mediastinal large B-cell lymphoma (PMLBCL). However, despite these advances, progressive disease (PD) remains the leading cause of treatment failure, with refractory patients exhibiting poor outcomes. The choice of salvage therapy following first-line treatment failure remains a critical factor in long-term survival [5].

In Iraq, previous studies have shown that Burkitt’s lymphoma (BL) is the most common type of non-Hodgkin lymphoma in children, accounting for a significant proportion of childhood cancers. A retrospective review in Iraq demonstrated that BL is responsible for approximately 40 % of all cases of malignant lymphomas in children under 14 years of age. The clinical and immunohistochemical profile of the disease has been established in Iraqi children, with the disease frequently presenting in the abdominal region, similar to patterns observed in other endemic areas. While treatment outcome research has been conducted for non-Hodgkin lymphoma in general, limited studies have focused specifically on the treatment outcomes and risk factors associated with BL in Iraq. For instance, one study noted a high mortality rate, with a significant portion of patients presenting with advanced-stage disease. The aggressive nature of BL, coupled with its association with Epstein-Barr virus and immunosuppression, emphasizes the need for further research and improved therapeutic strategies in the region [[4], [5], [6]].

In LMICs, pediatric B-NHL poses additional challenges due to limited diagnostic resources, inadequate supportive care, and high rates of treatment-related mortality. Studies have identified key risk factors associated with poor outcomes, including advanced-stage disease at diagnosis, presence of systemic symptoms, large tumor burden, and pleural effusions. Additionally, treatment at centers lacking the capacity to monitor tumor lysis syndrome (TLS) markers has been linked to increased early mortality. Addressing these challenges requires optimized risk stratification, early intervention strategies, and improved access to essential supportive care measures [7,8].

This study aims to retrospectively analyze a cohort of children and adolescents with B-NHL treated at Al-Hasan Al-Mojtaba Hospital over a 10-year period. The objectives include:

1. Assessing clinical characteristics, treatment regimens, and survival outcomes in pediatric B-NHL.
2. Comparing the effectiveness of the modified LMB96 regimen and the R-CHOP regimen in terms of survival and treatment-related complications.
3. Identifying prognostic factors associated with poor outcomes, with a focus on treatment failures and salvage therapy responses.

By addressing these objectives, this study aims to contribute to better treatment optimization and improved survival rates for pediatric B-NHL patients, particularly in LMICs, where outcomes remain significantly lower than in high-income settings.

## 2 Patients and methods

This retrospective study reviewed children and adolescents diagnosed with mature B-cell non-Hodgkin lymphoma (B-NHL) at Al-Hasan Al-Mojtaba Hospital between January 2014 and December 2024. Patients were treated with a modified LMB96 regimen, which was replaced by the R-CHOP regimen in the last two years of the study period. Clinical characteristics, treatment outcomes, and survival rates were analyzed. A comparative analysis was performed to evaluate the outcomes associated with the LMB96 and R-CHOP regimens.

### 2.1 Eligibility criteria

Only individuals with a confirmed diagnosis of Large B-cell lymphoma or Burkitt’s lymphoma were included in our study. Diagnosis confirmation was based on the WHO classification of lymphoid neoplasms, which requires either histological examination of biopsied specimens or cytologic examination of aspirates from affected lymph nodes or other tissues [9]. Individuals with a confirmed diagnosis but incomplete medical records, or those who received treatment for Burkitt’s lymphoma at other institutions, were excluded from the study sample.

Staging of the disease was performed according to the St. Jude staging system. This included physical examination, complete blood count with peripheral blood smear examination, bilateral bone marrow aspirates and biopsies, cerebrospinal fluid (CSF) analysis, and imaging using computed tomography (CT), magnetic resonance imaging (MRI), or positron emission tomography (PET), when affordable. Bone marrow involvement was defined as the presence of malignant lymphoma cells in a bone marrow smear [10]. Central nervous system (CNS) involvement was confirmed in the presence of at least one of the following: lymphoma cells identified in CSF cytology, intracranial or parameningeal lesions on radiological studies, cranial nerve palsy, or clinical spinal cord compression.

The staging system used is as follows:

• **Stage I**: A single tumor (extranodal) or a single anatomic area (nodal), excluding the mediastinum or abdomen, or a single extranodal tumor with regional node involvement.
• **Stage II**: Two or more nodal areas on the same side of the diaphragm, two single extranodal tumors with or without regional node involvement on the same side of the diaphragm, or a primary gastrointestinal tumor, typically in the ileocecal area, with or without involvement of associated mesenteric nodes, provided it is grossly completely resected.
• **Stage III**: Two single extranodal tumors on opposite sides of the diaphragm, two or more nodal areas above and below the diaphragm, all primary intra-thoracic tumors (mediastinal, pleural, thymic), extensive primary intra-abdominal disease, or paraspinal or epidural tumors, regardless of other tumor sites.
• **Stage IV**: Any of the above with initial CNS and/or bone marrow involvement.

Risk classification was also assessed using the FAB/LMB risk group classification:

• **Group A**: Patients with resected stage I and abdominal stage II disease only.
• **Group B**: Patients with non-resected stage I & II, stage III, or stage IV disease who are CNS negative and have bone marrow blasts < 25 %.
• **Group C**: Patients with CNS involvement and/or bone marrow blasts > 25 %.

### 2.2 Treatment procedure

In this study, patients were treated according to a modified protocol based on international clinical trials conducted by SFOP, CCG, and UKCCSG [11,12]. This modification aimed to reduce the short-term toxicity of methotrexate (Methotrexate) chemotherapy. A high dose of methotrexate (Methotrexate) was administered for groups B and C, which was lower than the original protocol doses (3 g/m² for group B and 8 g/m² for group C).

For **Group A**, treatment consisted of two cycles of COPAD. The specific regimen included:

• **Vincristine**: 2 mg/m² intravenously on Days 1 and 6
• **Prednisolone**: 60 mg/m²/day orally in two divided doses from Days 1–5, tapering to zero over three days
• **Cyclophosphamide**: 250 mg/m²/dose every 12 h as a 15-minute infusion on Days 1–3
• **Doxorubicin**: 60 mg/m² as a 6-hour infusion after the first dose of cyclophosphamide

Hydration was maintained at a rate of 3000 mL/m²/day (125 mL/m²/hr) and continued for 12 h after the last dose of cyclophosphamide. The interval between the first and second cycles of COPAD should not exceed 21 days.

For **Groups B and C**, treatment began with a reduction phase using COP. The regimen for both groups included:

• **Cyclophosphamide**: 300 mg/m² as an infusion over 15 min on Day 1
• **Vincristine**: 1 mg/m² intravenously on Day 1
• **Prednisolone**: 60 mg/m²/day orally in two divided doses from Days 1–7
• **Intrathecal methotrexate (Methotrexate)**: 8–15 mg according to age
• **Intrathecal hydrocortisone**: 8–15 mg according to age

For **Group B**, the intrathecal treatment was administered only on Day 1. For **Group C**, intrathecal methotrexate (Methotrexate) and hydrocortisone were given on Days 1, 3, and 5, with the addition of intrathecal cytarabine (Cytarabine).

Vigorous precautions were taken to minimize the risk of tumor lysis syndrome, especially for children with bulky disease in Groups B and C. Tumor response was evaluated on Day 7. A reduction of >20 % in tumor size was required before proceeding to COPADM. If the reduction was <20 %, the patient was reclassified to Group C.

After the reduction phase, patients received two cycles of COPADM, which included:

• **Cyclophosphamide**: 250 mg/m² every 12 h as a 15-minute infusion on Days 1–3
• **Vincristine**: 1 mg/m² intravenously on Day 1 for Group B, and 2 mg/m² for Group C
• **Prednisolone**: 60 mg/m²/day orally on Days 1–5
• **Doxorubicin**: 60 mg/m² as a 6-hour infusion after the first dose of cyclophosphamide
• **methotrexate (Methotrexate)**: 2 g/m² as a 3-hour infusion on Day 1 for Group B and 4-hour infusion for Group C
• **Folinic acid**: 15 mg/m² orally every 6 h for a total of 8 doses starting 24 h after the methotrexate (Methotrexate) infusion

For **Group C**, intrathecal chemotherapy included methotrexate (Methotrexate) and hydrocortisone on Days 2 and 6, with additional cytarabine (Cytarabine) on Days 1, 4, and 6.

Following the first cycle of **CYM** chemotherapy for Group B, response was reassessed. If any residual masses were detected, surgical excision or biopsy was performed. If histology was negative, the patient proceeded with the second cycle of CYM. If positive for malignant cells, the patient was reclassified to Group C and resumed treatment with **CYVE** chemotherapy.

For **Group C**, children with CNS involvement were treated with high-dose methotrexate (Methotrexate) and intrathecal chemotherapy following the first cycle of CYVE.

**Group C** patients continued with four courses of maintenance chemotherapy at 28-day intervals, consisting of:

• **Maintenance 1**: Vincristine, prednisolone, cyclophosphamide, methotrexate (Methotrexate), doxorubicin, and intrathecal chemotherapy
• **Maintenance 2 and 4**: cytarabine (Cytarabine) and etoposide (Etoposide)
• **Maintenance 3**: Vincristine, prednisolone, cyclophosphamide, and doxorubicin

Granulocyte-colony stimulating factor (G-CSF) at 5 mg/kg/day was administered starting 24 h after completing COPAM and CYVE chemotherapy until the post-nadir absolute neutrophil count (ANC) reached 3000/mm³.

In the last two years of the study, the chemotherapy protocol was changed to **R-CHOP** (Rituximab, cyclophosphamide, doxorubicin, vincristine, and prednisolone). The R-CHOP regimen included:

• **Rituximab**: 375 mg/m² on Day 1
• **Vincristine**: 1.4 mg/m² on Day 2
• **Doxorubicin**: 50 mg/m² on Day 2 as continuous infusion
• **Cyclophosphamide**: 750 mg/m² as continuous infusion on Day 2
• **Prednisolone**: 60 mg/m²/day for 5 consecutive days (Days 2–6)

For intrathecal chemotherapy, the protocol was adjusted depending on the group and CNS involvement.

## 3 Results

### 3.1 Sample characteristics

Medical records belonging to 66 patients were analyzed in our study; the median age of presentation was 5.8 years (range, 2.5–15 year), of those 46 (69.7 %) were male and 20 (30.3 %) were female. The most common histological type was BL (57, 86.3 %), followed by DLBL (9, 13.7 %). As shown in Table 1, the abdomen was the most common primary site of involvement in 56 patients (84.8 %) while genitourinary and spinal cord B-cell lymphoma was the least common and occurred in only one patient for each. In our sample, the majority of patients (48, 72.7 %) were present in advance stages of their illness (i.e., stages 3 and 4), and around two-thirds of the patients were assigned to group B (41, 62.1 %), followed by group C (19, 28.8 %). Isolated bone marrow (29) and CSF involvement was seen in 10 (15.1 %) patients for each, while patients who had combined BM and CSF involvement were 4 (6 %). By the end of the follow-up period, 19 (28.8 %) patients had suffered from a fatal outcome, the outcome status could not be established in 3 cases.

**Table 1:** Represents characterization of the study subjects.

Characteristics	*N* = 66 (%)
**Gender**	
Male	46 (69.7)
Female	20 (30.3)
**Residency**	
Kerbala	23 (34.8)
Outside of Kerbala	43 (65.2)
Babylon	14 (21.2)
Al-Qadisiyah	13 (19.7)
Najaf	7 (10.6)
Others	9 (13.6)
**Primary site of involvement**	
Abdomen	56 (84.8)
Head and neck	8 (12.2)
Genitourinary Spinal cord	1 (1.5) 1 (1.5)
**Site specification**	
Ileocecal	50 (75.7)
Cervical lymph nodes	4 (6.0)
Pleuro-peritoneal nodular thickening	1 (1.5)
Porta hepatis	2 (3.0)
Right colon	1 (1.5)
Para-aortic lymph nodes	1 (1.5)
Tonsillar	1 (1.5)
Testicular	1 (1.5)
Posterior nasal Nasopharynx Submandibular Thoracic segments of spinal cord Testis	1 (1.5) 1 (1.5) 1 (1.5) 1 (1.5) 1 (1.5)
**Bone Marrow involvement**	
Positive	10 (15.2)
Negative	56 (84.8)
**CSF Involvement**	
Yes	10 (15.2)
No	56 (84.8)
**Staging**	
I	3 (4.5)
II	15 (22.7)
III	31 (47.0)
IV	17 (25.8)
**Grouping**	
Group A	6 (9.1)
Group B	41 (62.1)
Group C	19 (28.8)
**Event**	
None	43 (65.1)
Progression	4 (6.1)
Recurrence	1 (1.5)
Abandonment	4 (6.1)
Death	14 (21.2)
**Outcome**	
Death	19 (28.8)
Alive	36 (66.6)
Unknown	2 (3.8)

### 3.2 Gender and disease characteristics

Table 2 summarizes the distribution of disease characteristics according to patients’ gender. All female patients included in our study have an abdominal primary site of involvement compared to 32 (82.1 %) male patients. Female patients were also more likely to have bone marrow involvement (33.3 % vs. 12.8 %) and less likely to have CSF involvement (6.7 % vs. 17.9 %). However, none of these differences reached statistical significance.

**Table 2:** Association between patient’s gender and disease characteristics.

Characteristic	Male (%)	Female (%)	P-value
**Primary site of involvement**			
Abdominal	37 (66.1)	19 (33.9)	0.09
Head and neck	8 (12.1)	0 (0.0)	
Genitourinary Spinal cord	1 (1.5) 1 (1.5)	0 (0.0) 0 (0.0)	
			
**Bone Marrow involvement**			
Positive	5 (7.6)	5 (7.6)	0.08
Negative	42 (63.6)	14 (21.2)	
**CSF Involvement**			
Yes	9 (13.6)	1 (1.5)	0.08
No	38 (57.6)	18 (27.3)	
**Staging**			
I	3 (4.5.0)	0 (0.0)	0.19
II	11 (16.7)	3 (4.5)	
III	20 (30.3)	11 (16.7)	
IV	12 (15.2)	5 (7.6)	
**Grouping**			
Group A	4 (6.1)	2 (3.0)	0.25
Group B	31 (47.0)	10 (15.2)	
Group C	12 (18.2)	7 (10.6)	
**Event**			
None	29 (43.9)	14 (21.2)	0.91
Progression	4 (6.1)	0 (0.0)	
Recurrence	1 (1.5)	0 (0.0)	
Abandonment	3 (4.5)	1 (1.5)	
Death	10 (15.2)	4 (6.1)	
**Outcome**			
Death	15 (22.7)	4 (6.1)	0.61
Alive	29 (43.9)	15 (22.7)	
Unknown	2 (3.0)	1 (1.5)	

### 3.3 Staging, grouping, and disease outcome

The association between each of the disease staging and patient grouping is simplified in Table 3, Table 4, respectively. No statistically significant association was demonstrated between grouping and staging with disease outcome.

**Table 3:** Association between staging and disease outcome.

Outcome	Stage I (%)	Stage II (%)	Stage III (%)	Stage IV (%)	P-value
**Clinical event**					
None	2 (3.0)	13 (19.7)	21 (31.8)	7 (10.6)	0.14
Progression	1 (1.5)	0 (0.0)	1 (1.5)	2 (3.0)	
Recurrence	0 (0.0)	1 (1.5)	0 (0.0)	0 (0.0)	
Abandonment	0 (0.0)	0 (0.0)	2 (3.0)	2 (3.0)	
Death	0 (0.0)	1 (1.5)	7 (10.6)	6 (9.1)	
**Outcome**					
Death	1 (1.5)	2 (3.0)	8 (12.1)	8 (12.1)	0.12
Alive	2 (3.0)	13 (19.7)	22 (33.3)	7 (10.6)	
Unknown	0 (0.0)	0 (0.0)	1 (1.5)	2 (3.0)

**Table 4:** Association between grouping and disease outcome.

Outcome	Group A (%)	Group B (%)	Group C (%)	P-value
**Clinical event**				
None	6 (9.1)	28 (42.4)	9 (13.6)	0.5
Progression	0 (0.0)	2 (3.0)	2 (3.0)	
Recurrence	0 (0.0)	1 (1.5)	0 (0.0)	
Abandonment	0 (0.0)	2 (3.0)	2 (3.0)	
Death	0 (0.0)	8 (12.1)	6 (9.1)	
**Outcome**				
Death	0 (0.0)	11 (16.7)	8 (12.1)	0.22
Alive	6 (9.1)	29 (43.9)	9 (13.6)	
Unknown	0 (0.0)	1 (1.5)	2 (3.0)	

### 3.4 Event-Free survival by disease grouping

**Fig. 1:**
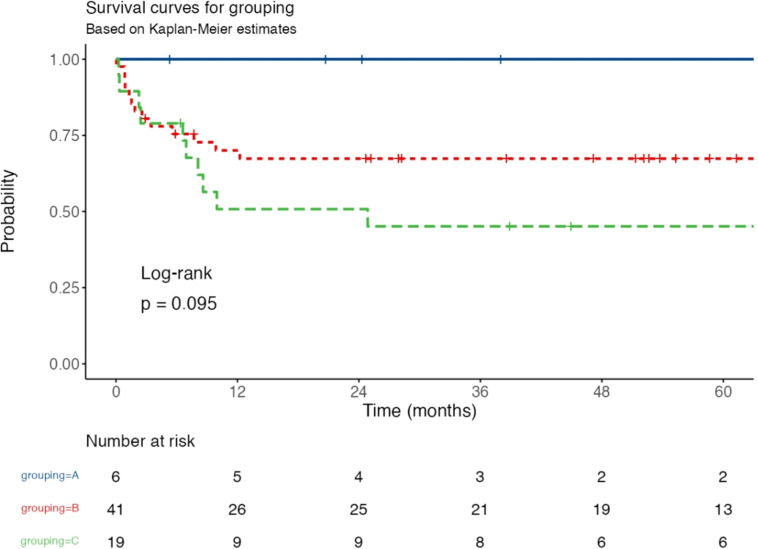
Kaplan-Meier Survival Curves for Event-Free Survival (EFS) by Disease Grouping. EFS over five years for Group A (blue, resected early-stage disease), Group B (red, advanced but CNS-negative disease), and Group C (green, CNS involvement and/or high bone marrow blasts). Group A had the best prognosis, while Group C showed the lowest survival. The log-rank test yielded *p* = 0.095, indicating a trend toward significance. The table below shows the number of patients at risk over time.

A five-year Kaplan-Meier survival analysis was conducted to evaluate event-free survival (EFS) among three disease groups: Group A (patients with resected stage I and abdominal stage II disease), Group B (patients with non-resected stage I & II, stage III, or stage IV disease who are CNS-negative with <25 % bone marrow blasts), and Group C (patients with CNS involvement and/or >25 % bone marrow blasts). The survival curves (Fig. 1) showed that Group A had the most favorable prognosis, maintaining an EFS close to 100 % throughout the study period. Group B experienced a moderate decline, stabilizing around 75 %, while Group C had the poorest outcomes, with a steep drop in EFS within the first 12 months, reaching approximately 50 % thereafter. A log-rank test comparing survival distributions yielded a p-value of 0.095, suggesting a trend toward statistical significance, though not reaching the conventional threshold (*p* < 0.05). The number at risk table reflects the declining sample sizes over time, particularly in Groups B and C, which had higher attrition rates due to events.

### 3.5 Survival curve by staging

**Fig. 2:**
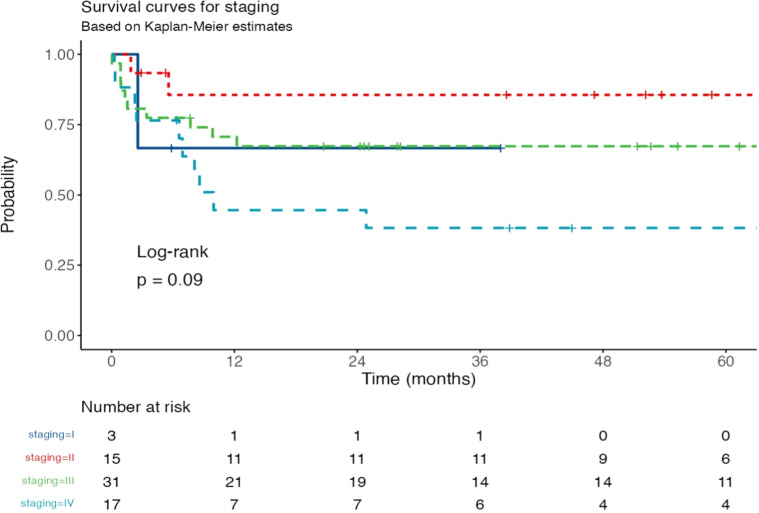
Kaplan-Meier survival curves for event-free survival (EFS) based on disease staging. The survival probabilities are plotted over time (in months) for Stage I (solid blue line), Stage II (dotted red line), Stage III (dashed green line), and Stage IV (dashed light blue line). The number at risk for each stage is displayed below the x-axis at different time points. The log-rank test yielded *p* = 0.021, indicating a statistically significant difference in survival among the stages. Patients in Stage II exhibited the highest survival probability, while those in Stage IV had the poorest outcomes, with a steep decline in survival early in the follow-up period.

The Kaplan-Meier survival analysis demonstrated significant differences in event-free survival (EFS) among different disease stages (*p* = 0.021, log-rank test, Fig. 2). Stage II patients (dotted red line) exhibited the highest survival probability, with only a slight decline over time. Stage III patients (dashed green line) showed an intermediate survival trend, with a gradual decrease in probability. Stage I patients (solid blue line) had a small sample size, making direct comparisons challenging, but their survival probability remained relatively stable. In contrast, Stage IV patients (dashed light blue line) had the poorest outcomes, with a steep decline in survival probability early on. The number at risk table indicates that at 60 months, 6 Stage II, 10 Stage III, and 4 Stage IV patients remained at risk, while no Stage I patients were at risk beyond 48 months. These findings suggest that disease stage significantly influences survival, with more advanced stages associated with worse prognosis.

### 3.6 Survival curve by genders

**Fig. 3:**
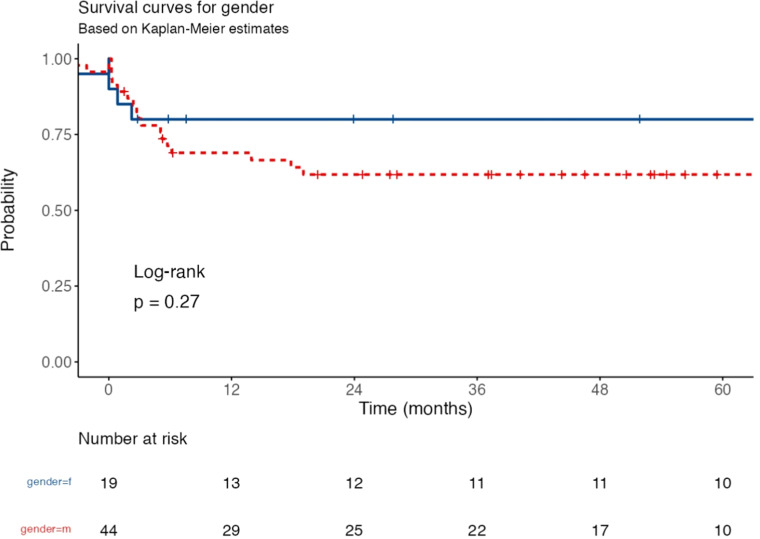
Kaplan-Meier survival curves for event-free survival (EFS) based on gender. The survival probabilities are plotted over time (in months) for **females (solid blue line) and males (dotted red line)**. The number at risk for each group is displayed below the x-axis at different time points. The log-rank test yielded ***p* = 0.27**, indicating no statistically significant difference in survival between genders. While females showed a higher survival probability throughout the follow-up period, the difference was not significant.

The Kaplan-Meier survival analysis compared event-free survival (EFS) between genders over time (Fig. 3), showing a trend where females (solid blue line) had higher survival probabilities compared to males (dotted red line). The survival probability for females remained consistently high throughout the follow-up period, whereas males exhibited a gradual decline in survival over time. The number at risk table demonstrated that at 60 months, 10 females and 10 males remained at risk. Despite the observed difference in survival curves, the log-rank test yielded a p-value of 0.27, indicating that the difference was not statistically significant. These findings suggest a potential trend of better survival outcomes in females, but further investigation with a larger sample size may be needed to confirm this observation.

## 4 Discussion

Mature B-cell lymphoma is an aggressive type of NHL in children. Numerous studies have been conducted to optimize treatment and improve outcome [13,14]. In spite of spectacular improvement in outcome in high income countries, prognosis is still disappointing in low- and middle-income nations [[15], [16], [17]]

Our retrospective study reviewed the clinical characteristics of patients, their outcomes according to risk stratifications and treatment regimen. In this study, more than two-third of the patients were male (69.7 %), this gender predominance is consistent with previous studies examining the demographic and clinical characteristics of pediatric patients with Burkitt’s lymphoma [18,19]. In terms of gender differences in clinical presentation, bone marrow involvement was equal in both genders, while CSF involvement was more common in males (13.6 % vs 1.5 %), however, this difference did not reach statistical significance presumably due to small sample size. Central nervous system involvement in Burkitt’s lymphoma is considered a bad prognosis sign causing complicated treatment challenges [20,21], previous studies have estimated the prevalence of CNS involvement at 5 % - 40 % at presentation. in our study ten patients (15.2 %) had CSF involvement, demonstrating relatively higher incidence of CNS involvement in our cohort of patients which may be related to delay in seeking medical advice and diagnosis.

Most children presented with abdominal involvement (84.8 %), Previous studies from Uganda and Kenya have found that most patients presented with facial tumors followed by abdominal sites [22,23]. Nineteen patients died due to B-cell lymphoma during the study period, resulting in a mortality rate of 28.8 %. In comparison, the five-year survival rate for children with Burkitt’s lymphoma in the USA was around 90.4 %, while children from Sub-Saharan Africa had only a 60 % survival rate [24].

At presentation, most patients were in advanced stage (i.e., stage III and IV, *n* = 48, 72.7 %), and about one third (28.8 %) were assigned to group C, having either bone marrow or CSF involvement (or both). This is a concerning finding as previous studies have demonstrated that patients presenting while in an advanced stage of their illness carry a higher mortality rate [24]; considerable number of patients with advanced stage may account for low outcome in our study. However, small sample sizes in individual groups have the potential to undermine the significance of certain survival trends due to the higher impact of the chance effect. In this study, a high dose of methotrexate (Methotrexate) was applied as 2gm/m^2^ for groups B and C, which is lower than what has been used in the original protocol (3gm/m^2^ for group B, and 8gm/m^2^ for group C); as the OS and EFS between disease grouping did not reach statistical significance (i.e., P-value 0.14, and 0.095, respectively), this may indicate the possibility of further waiving of chemotherapy in advanced B-cell lymphoma with a consequent decrease in chemotherapy side effects.

Rituximab, which is a monoclonal antibody with a high affinity against CD20 in B cells has been reported to provide a good outcome when combined with chemotherapy. In adults with B-NHL, and due to its demonstrated effectiveness, rituximab is now a routine part of treatment [25,26]. Goldman et al. [27,28]; Mitchell S. Cairo proposed that addition of rituximab to the standard LMB96 protocol in pediatric patients with BL who had BM (≥25 % blasts) or CNS involvement would be safer and more effective [29,30]. The efficacy of rituximab in high-risk patients classified as group B was also supported by Goldman *et al*. [29,30]. In this study, patients with stage III/IV and group B mature B-NHL who received rituximab with the FAB/LMB96 protocol showed an improvement in the 3-year EFS rate (95 % in the R-LMB group), without serious adverse events

Minard-Colin *et al*. [31] compared the R-LMB protocol with the standard LMB protocol alone in high-risk (stage III or stage IV) patients with mature B-NHL; they reported a markedly improved outcome in the R-LMB group. On the basis of these reports, the NCCN guideline recommended rituximab-combined chemotherapy in high-risk patients classified as groups B and C [32].

In our study, many patients underwent therapy with the combination of rituximab and the CHOP protocol, all showed good treatment outcomes without treatment-related complications.

Taking into consideration that a significant percentage of mortality in our cohort patients was due to chemotherapy side effects (especially high dose methotrexate) and infections (because of the delay in seeking medical advice), one can assume that good outcome of advanced B cell lymphoma can be achieved with the lower dose of chemotherapy. The low incidence of recurrent or relapse states in this study can further support the above-mentioned assumption.

In our study, the OS and EFS rates in our patients were 68.2 % and 63.5 %, respectively. There was no significant statistical difference of the EFS according to staging (p-value, 0.09), grouping (p-value, 0.095), and treatment regimen (p-value, 0.25). Although the outcome is still low when compared with other published studies in high income nations, one can address the importance of selecting less intensive chemotherapy combined with rituximab in low- and middle-income nations to avoid toxic death related chemotherapy as well as infection-related sequelae during the period of neutropenia. Although the outcome is still low when compared with other published studies in high income nations, one can address the importance of selecting less intensive chemotherapy combined with rituximab in low- and middle-income nations to avoid treatment-related toxicity and infection-related sequalae which remain important concerns in LMIC.

Moreover, the role of high dose methotrexate (Methotrexate) in advanced disease (i.e., group B and C) was refuted in this study as lower dose of methotrexate (Methotrexate) was given (2 g/m^2^) without significant influence at the outcome. The main limitation of this study was the small sample of patients as it was a single center study. Multicenter study should be encouraged especially in LMIC nations so as to establish a guideline for treatment of children with B-cell lymphoma in LMIC nations. In summary, this study may highlight the importance of reducing the intensity of chemotherapy in children with B-cell lymphoma especially when there is good initial response, applying a rituximab-based chemotherapy regimen, and establishing a guideline for treatment in LMIC nations.

## 5 Conclusion

This study provides valuable insights into the clinical characteristics, treatment outcomes, and challenges associated with pediatric aggressive mature B-cell lymphoma in a resource-limited setting. The findings highlight the high prevalence of advanced-stage disease at presentation, which contributes to the lower survival outcomes compared to high-income countries. Despite the use of reduced-intensity chemotherapy with lower-dose methotrexate (Methotrexate), survival outcomes did not significantly differ across staging, grouping, or treatment regimens, suggesting the potential for chemotherapy de-escalation to minimize toxicity while maintaining efficacy. The addition of rituximab to standard chemotherapy regimens demonstrated promising results, reinforcing its role in improving event-free survival in high-risk patients. However, treatment-related mortality due to chemotherapy toxicity and infections remains a major concern, emphasizing the need for optimized supportive care strategies. The study underscores the importance of developing tailored treatment guidelines for low- and middle-income countries to balance efficacy with toxicity. Larger, multicenter studies are necessary to further refine treatment protocols and improve outcomes for children with B-cell lymphoma in these settings.

## Ethical considerations

To ensure patient confidentiality, de-identified patient data was used during the analysis and reporting of results in our study. Ethical approval was obtained from the Institutional Review Board of the Ministry of Health (MOH)/Kerbala directorate in 2014 prior to data collection.

## CRediT authorship contribution statement

**Hamid D. Habeeb Rjeib:** Conceptualization, Formal analysis, Investigation, Project administration, Software, Validation, Writing – original draft, Data curation, Funding acquisition, Methodology, Resources, Supervision, Visualization, Writing – review & editing. **Usama Al-Jumaily:** Investigation, Project administration, Software, Visualization, Conceptualization, Methodology, Resources, Validation, Writing – original draft. **Inas Muayad Mohammed Ali:** Data curation, Investigation, Project administration, Validation, Conceptualization, Formal analysis, Methodology, Resources. **Safa Faraj:** Investigation, Project administration, Validation, Methodology, Software, Visualization, Writing – review & editing, Writing – original draft. **Mohammed Fawzi Al-Qanbar:** Data curation, Investigation, Validation, Formal analysis, Methodology, Visualization. **Dheyaa Aldeen Al-Khateeb:** Software, Validation, Writing – original draft, Resources, Supervision, Visualization. **Shaima Jabbar:** Methodology, Validation, Writing – original draft, Formal analysis, Supervision, Visualization.

## Declaration of competing interest

The authors declare that they have no known competing financial interests or personal relationships that could have appeared to influence the work reported in this paper.
